# The Love of Man to Man

**Published:** 1889-08-17

**Authors:** 


### Words of Consolation.

### THE LOVE OF MAN TO MAN.

(*For Reading to the Sick.*)

Our Lord's life on earth was the perfect pattern of what we should aim at in our own case. "He went about doing good," and, although we may not be called upon to give up every home tie and devote ourselves entirely to relieve the bodily and spiritual wants of our fellow-creatures, yet we should try as far as possible to make our lives resemble His. It was His intense sympathy with man which drew Him down from His glory in heaven to be our benefactor for time and eternity, and we should foster this sympathy in ourselves, and throw it over all near and dear to us, until, like a stone cast into the water, the circles widen and widen, embracing at last every soul for whom Christ died. This sympathy will not be unaccompanied by pain. All true sympathy *is* pain, for is it not lightening other people's trials by bearing them ourselves? And as our dear Lord bore the pain of His sympathy in hunger and thirst, in faintness and weariness, in grief, for the hardness of men's hearts, so must we be prepared to follow in His footsteps for weal and woe. St. James tells us "pure religion and undefiled" is "to visit the fatherless and widows in their afflictions and to keep ourselves from the world." The Galatians received the command from St. Paul, "Bear ye one another's burdens, and so fulfil the law of Christ," and in his first Epistle to the Corinthians he enlarges on what charity, or love, would do or leave undone. Without it, he says, we are but as "sounding brass or a tinkling cymbal"—that is, our good works are worthless when done with show and noise to attract attention to them. Then he shows us what the man is like who is full of charity, and zeal, and love for God and his neighbour. He suffers long and is kind to those who afflict him, he envieth not any richer in health or wealth than himself; he does not boast nor is he vain of his attainments, he behaves seemly—that is, becomingly wherever or with whomsoever he may be—he even forbears to claim his own, he is not easily provoked, he hateth wickedness and lies, but rejoiceth in the truth; "he beareth all things, hopeth all things, endureth all things." On these lines, as has been well said, may be formed the "finest of fine manners," pleasing alike to God and man.

And now how shall we carry out these evident duties? By beginning at home. "The daily round, the common task" will find plenty of "room to deny ourselves," and this is a comforting thought to those whose household duties and family cares prevent their going beyond their own doorstep to seek ways of usefulness, for the home life can be sweetened and made bright and cheerful by the "soft answer which turneth away wrath," by the kindly word and the helpful deed, and by believing in the good motives of friends and dependents, even when their actions are impatient or unkind. "Blessed are the peace makers!" But to those whose time is comparatively their own numberless ways present themselves of "bearing one another's burdens"—the willing mind, the ready wit, and the gentle hand, may be used to tend the bodies racked with pain or tossed with fever frenzy; the hungry, the naked, the ignorant, may be sought for and be fed, and clothed, and taught; and always bearing in mind St. Peter's caution—"be pitiful, be courteous"—we should try to find out and understand the hidden griefs and sorrows which bow down many hearts, and, by loving, gentle words, to soothe and calm them.

